# Oral Lysozyme Attenuates Neuroinflammation and Brain Injury After Traumatic Brain Injury Through Gut Microbiota‐Dependent Reprogramming of Tryptophan Metabolism

**DOI:** 10.1002/cns.71025

**Published:** 2026-07-10

**Authors:** Zhuoying Du, Haijun Yao, Caihua Xi, Qiang Yuan, Pengfei Fu, Jin Hu, Gang Wu, Weijian Yang

**Affiliations:** ^1^ Department of Neurosurgery, Huashan Hospital, Shanghai Medical College Fudan University Shanghai China; ^2^ National Center for Neurological Disorders Shanghai China; ^3^ Shanghai Key Laboratory of Brain Function Restoration and Neural Regeneration Shanghai China; ^4^ Neurosurgical Institute of Fudan University Shanghai China; ^5^ Shanghai Clinical Medical Center of Neurosurgery Shanghai China; ^6^ Department of Neurosurgery and Neurocritical Care, Huashan Hospital, Shanghai Medical College Fudan University Shanghai China

**Keywords:** gut–brain axis, kynurenic acid, lysozyme, neuroinflammation, traumatic brain injury, tryptophan metabolism

## Abstract

**Aims:**

Traumatic brain injury (TBI) induces secondary neuroinflammation and gut dysbiosis. This study investigated whether oral lysozyme confers neuroprotection after TBI through gut microbiota‐dependent metabolic reprogramming of tryptophan metabolism.

**Methods:**

In a severe TBI mouse model, neurological function, neuroinflammation, intestinal barrier integrity, and systemic immune homeostasis were assessed following oral lysozyme administration. Fecal untargeted metabolomics, antibiotic‐mediated microbiota depletion, and fecal microbiota transplantation (FMT) were used to explore microbiota involvement. Cerebrospinal fluid (CSF) from 10 matched pairs of patients with severe TBI was analyzed for tryptophan pathway metabolites by liquid chromatography–mass spectrometry.

**Results:**

Lysozyme improved neurological outcomes, attenuated neuronal apoptosis and neuroinflammation, and restored peripheral CD4^+^/CD8^+^ T cell homeostasis. Metabolomics revealed enrichment of fecal tryptophan metabolites (indole‐3‐carboxaldehyde, indolelactic acid, kynurenic acid [KYNA]) and a shift in cerebral kynurenine metabolism toward the KYNA branch. These associations were abolished by microbiota depletion and reproduced by FMT. Favorable clinical outcomes were associated with higher CSF KYNA and an elevated KYNA/QA ratio.

**Conclusions:**

Oral lysozyme was associated with attenuated TBI‐induced neuroinflammation and brain injury, potentially through gut microbiota‐dependent tryptophan metabolism reprogramming. Concordance between preclinical and clinical metabolomic data supports lysozyme as a candidate microbiota‐targeted therapeutic strategy. The KYNA/QA ratio warrants further validation as a prognostic indicator in larger, longitudinal cohorts.

## Graphical abstract

Oral lysozyme reshapes gut microbiota‐dependent tryptophan metabolism, increasing neuroprotective metabolites like KYNA and restoring intestinal barrier integrity. This attenuates systemic inflammation and reprograms cerebral kynurenine metabolism toward the neuroprotective KYNA branch, reducing neuroinflammation and improving outcomes after traumatic brain injury.

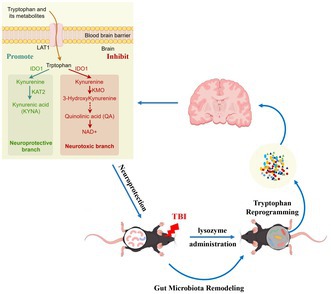

**Abbreviations**
3‐HK: 3‐hydroxykynurenine
AhR: aryl hydrocarbon receptor
CNS: central nervous system
CSF: cerebrospinal fluid
ELISA: enzyme‐linked immunosorbent assay
FMT: fecal microbiota transplantation
GCS: Glasgow Coma Scale
GOS: Glasgow Outcome Scale
I3A: indole‐3‐carboxaldehyde
IDO1: indoleamine 2,3‐dioxygenase 1
IL: interleukin
ILA: indolelactic acid
KAT‐II: kynurenine aminotransferase II
KMO: kynurenine 3‐monooxygenase
KYNA: kynurenic acid
LAT1: L‐type amino acid transporter 1
LC–MS: liquid chromatography–mass spectrometry
mNSS: modified neurological severity scores
QA: quinolinic acid
SPF: specific pathogen‐free
TBI: traumatic brain injury
TNF: tumor necrosis factor
Trp: tryptophan
ZO‐1: zonula occludens‐1

## 1 Introduction

Traumatic brain injury (TBI) remains one of the most prevalent causes of mortality and persistent neurological impairment globally, with a disproportionate burden on the young adult population [1, 2]. Beyond the primary biomechanical injury, TBI triggers an extended secondary pathological cascade marked by neuroinflammatory activation, immune homeostasis disruption, metabolic dysregulation, and gradual neuronal attrition. Unfolding over a timeframe ranging from hours to weeks post‐injury, these secondary mechanisms offer a pivotal opportunity for therapeutic intervention [3]. The gut–brain axis has emerged as an important regulator of post‐traumatic neuroinflammation through neural, endocrine, immune, and metabolic pathways [4, 5]. TBI rapidly disrupts intestinal barrier integrity and induces gut dysbiosis [5, 6], facilitating the translocation of bacteria, microbial products, and metabolites that can amplify systemic inflammation and worsen central nervous system injury [7, 8]. Thus, the gut microbiota may represent a therapeutic target in TBI.

Within the spectrum of microbially generated mediators, the metabolic fate of tryptophan (Trp) holds distinct importance for central nervous system stability [9, 10]. Dietary Trp undergoes microbial conversion into bioactive indole derivatives—including indole‐3‐carboxaldehyde (I3A) and indolelactic acid (ILA)—that serve as ligands for the aryl hydrocarbon receptor (AhR), thereby supporting epithelial barrier function and immune homeostasis [11, 12, 13]. The kynurenine pathway constitutes the principal route of host Trp catabolism, and the ratio of neuroprotective kynurenic acid (KYNA) to neurotoxic quinolinic acid (QA) within this pathway serves as a critical determinant of neuroinflammation and neuronal fate [14]. Dysregulated microbial and kynurenine metabolism has been implicated in multiple neurological disorders [15], suggesting that modulation of gut microbiota toward a neuroprotective metabolic state may benefit TBI patients.

Produced and secreted by intestinal Paneth cells, lysozyme is a constitutive component of the innate immune arsenal that degrades bacterial peptidoglycan and contributes critically to gut microbial homeostasis [16, 17]. Our previous study showed that oral lysozyme alleviates intestinal injury after severe TBI by modulating gut microbiota composition and restoring barrier function [18]. However, whether lysozyme exerts remote neuroprotective effects through microbiota‐dependent metabolic reprogramming, particularly of the tryptophan pathway, remains unknown. Given the emerging translational importance of the gut–brain axis [19], we hypothesized that oral lysozyme reshapes gut microbiota metabolic output, thereby strengthening intestinal barrier integrity, restoring systemic immune homeostasis, and redirecting cerebral tryptophan metabolism toward a neuroprotective state. To test this hypothesis, we combined metabolomic profiling, microbiota depletion, fecal microbiota transplantation (FMT), and clinical cerebrospinal fluid (CSF) metabolomics to delineate the mechanistic link between lysozyme, gut microbiota metabolism, and neuroprotection after TBI.

## 2 Materials and Methods

### 2.1 Mice and Housing

To eliminate confounding by sex‐related hormonal influences, only male mice were used. Male C57BL/6 mice aged 6–8 weeks were maintained under specific pathogen‐free conditions. All mice underwent a 7‐day acclimation period before any experimental manipulation. Animals were maintained under controlled conditions (23°C ± 2°C, 12‐h light/dark cycle) and allowed free access to standard chow and sterilized drinking water. Cages and bedding were changed every 3 days. All experimental procedures complied with the relevant guidelines for animal research reporting [20].

### 2.2 Experimental Design and Severe TBI Model

Mice were randomly assigned to four groups using a computer‐generated randomization sequence: normal control (N), sham‐operated (S), severe TBI (T), and severe TBI + lysozyme treatment (L). Severe TBI was induced as previously described [21]. Lysozyme (Sigma‐Aldrich, USA) was administered to mice in the L group by oral gavage at a dose of 200 U/day for seven consecutive days starting from the day of TBI induction [22]. Mice in the N, S, and T groups received an equivalent volume of sterile drinking water via oral gavage over the same treatment period.

### 2.3 Antibiotic‐Mediated Microbiota Depletion and Fecal Microbiota Transplantation

To assess the microbiota dependence of lysozyme‐mediated protection, mice received an antibiotic cocktail (ABX; ampicillin 0.1 mg/mL, streptomycin 0.5 mg/mL, colistin 0.1 mg/mL) to deplete the microbiota [23]. These mice were randomly assigned to the following groups using a computer‐generated randomization sequence: ABX + sTBI + sterile water (AS), ABX + sTBI + lysozyme (ASL), and ABX + sTBI + FMT (AST). AST recipient mice received fecal microbiota from lysozyme‐treated donors.

### 2.4 Sample Collection

On Day 7, mice were anesthetized with sodium pentobarbital and euthanized by exsanguination. Blood, distal ileum, and brain tissues were snap‐frozen at −80°C or fixed in 4% paraformaldehyde for subsequent analyses. Ileocecal contents were stored at −80°C for metabolomic analysis.

### 2.5 Neurological Assessment

Neurological function was assessed using the modified Neurological Severity Score (mNSS) [24], comprising motor, sensory, reflex, and balance tests (range 0–18; higher scores indicate greater impairment). All assessments were performed by two investigators blinded to group allocation.

### 2.6 Flow Cytometry

Peripheral blood samples were collected for flow cytometric analysis. Fluorophore‐conjugated antibodies targeting CD3, CD4, and CD8a, together with their corresponding isotype controls, were obtained from BioLegend. Stained cells were analyzed using a flow cytometer (BD Biosciences), and the resulting data were processed with FlowJo software (version 10.0.7). Flow cytometry data were analyzed in a blinded manner using coded samples.

### 2.7 Enzyme‐Linked Immunosorbent Assay (ELISA)

Blood was collected via cardiac puncture into ethylene diamine tetraacetic acid (EDTA)‐precoated syringes. Plasma was then isolated by centrifugation at 1500 × *g* for 15 min at 4°C and subsequently stored at −80°C until analysis. Plasma levels of interleukin (IL)‐1β, tumor necrosis factor (TNF)‐α, IL‐6, IL‐10, and IL‐22 were measured using ELISA kits (R&D Systems, USA) according to the manufacturer's instructions. Data were analyzed in a blinded manner using coded samples.

### 2.8 Western Blotting

Cleaved caspase‐3 (brain) and ZO‐1 (distal ileum) expression were assessed by Western blotting. Tissue lysates were prepared using radio immunoprecipitation assay buffer (Beyotime, China), and protein concentrations were measured before loading. Fifty micrograms of protein per lane were separated on 12% sodium dodecyl sulfate–polyacrylamide gels and transferred to polyvinylidene fluoride membranes (Millipore, USA). Membranes were blocked with 1% nonfat milk in Tris‐buffered saline containing Tween 20 for 2 h at room temperature, then incubated overnight at 4°C with primary antibodies targeting cleaved caspase‐3 (Cell Signaling Technology, USA), β‐actin (Abcam, USA), and zonula occludens‐1 (Invitrogen, USA). After washing, membranes were incubated with horseradish peroxidase‐conjugated secondary antibodies for 1 h at room temperature. Immunoreactive bands were visualized using enhanced chemiluminescence detection (Thermo Fisher Scientific, USA). Densitometry was performed by two independent blinded investigators.

### 2.9 Quantitative Reverse Transcription‐Polymerase Chain Reaction (RT‐PCR)

Total ribonucleic acid was extracted from brain tissue and reverse‐transcribed into complementary deoxyribonucleic acid. Quantitative RT‐PCR was performed with an initial denaturation at 95°C for 5 min, followed by 40 cycles at 95°C for 15 s and 60°C for 30 s. Gene expression levels were analyzed for glyceraldehyde 3‐phosphate dehydrogenase (GAPDH), L‐type amino acid transporter 1 (LAT1), indoleamine 2,3‐dioxygenase 1 (IDO1), kynurenine monooxygenase (KMO), and kynurenine aminotransferase II (KAT‐II). Primer sequences were as follows: m‐glyceraldehyde 3‐phosphate dehydrogenase (GAPDH)‐F ACCCTTAAGAGGGATGCTGC, m‐GAPDH‐R CCCAATACGGCCAAATCCGT, m‐L‐type amino acid transporter 1 (LAT1)‐F GCTGACGAACCTGGCCTATT, m‐LAT1‐R TAGTTCCCGAAGTCCACAGC, m‐indoleamine 2,3‐dioxygenase 1 (IDO1)‐F CCAGTGCAGTAGAGCGTCAA, m‐IDO1‐R TCTGGGTCCACAAAGTCACG, m‐Kynurenine monooxygenase (KMO)‐F TGGAGTCCTATGCCAATGCG, m‐KMO‐R AACCTTGTCAGGTCCGAGC, m‐Kynurenine aminotransferase (KAT) II‐F TGGTTCAGCTCCTACCTCCT, m‐KAT II‐R CAATCTCTGGAAGGCTGTATCC.

### 2.10 Histological Analysis

Distal ileum and brain tissues were fixed in 4% paraformaldehyde, dehydrated through graded ethanol, embedded in paraffin, and sectioned at 5 μm. After deparaffinization and rehydration, sections were stained with hematoxylin and eosin (Beyotime, China). Histopathological changes were examined under a light microscope (Olympus, Japan). Intestinal injury was scored using a modified Chiu's system [25] (0–9 scale; 0 = normal, 9 = hemorrhage and ulceration). At least five randomly selected fields per specimen were scored, and the mean value was used. Scoring was performed by two independent blinded investigators.

### 2.11 Terminal Deoxynucleotidyl Transferase dUTP Nick‐End Labeling (TUNEL) Assay and Immunofluorescence Staining

Cell apoptosis in brain and ileal tissues was detected using an In Situ Cell Death Detection Kit (Roche, Germany). Paraffin‐embedded sections were baked at 60°C for 1 h, followed by deparaffinization and rehydration according to the manufacturer's protocol. Cell nuclei were counterstained with 4′,6‐diamidino‐2‐phenylindole (DAPI) (Beyotime, China). The number of TUNEL‐positive cells was quantified with ImageJ software (version 1.8.0_345). For zonula occludens‐1 (ZO‐1) immunofluorescence staining, ileal sections were incubated with anti‐ZO‐1 primary antibody (Invitrogen, USA) at 4°C overnight, then labeled with goat anti‐rabbit IgG H&L secondary antibody (Abcam, USA). Staining intensity was quantified as average optical density (AOD), calculated by dividing integrated optical density by the measured area. Quantification was performed by two independent blinded investigators.

### 2.12 Paired CSF Samples From Patients With Severe TBI

For clinical metabolomic analysis, we prospectively enrolled 10 pairs of patients with severe TBI matched for age, sex, injury mechanism, time from injury to sampling, Glasgow Coma Scale (GCS) score, and GCS motor score. Eligibility required age ≥ 18 years, first admission for severe TBI defined as a Glasgow Coma Scale score ≤ 8, and presentation within 24 h of injury. Patients were excluded if they met any of the following criteria: (1) pregnancy; (2) preexisting central nervous system disease, immunodeficiency, diabetes mellitus, or coagulation disorders; (3) significant hepatic or renal impairment; (4) immunosuppressive or anti‐inflammatory therapy at the time of admission; or (5) loss to follow‐up or incomplete clinical data. Each pair comprised one patient with a favorable and one with an unfavorable outcome, defined by the Glasgow Outcome Scale (GOS) at 6 months after discharge. Baseline characteristics are summarized in Table S1. CSF samples were collected during intraventricular catheter insertion for ventricular drainage or intracranial pressure monitoring within 24 h of injury, processed, and stored at −80°C within 30 min.

### 2.13 Metabolomic Profiling

Untargeted metabolomic profiling was conducted using a high‐resolution liquid chromatography–mass spectrometry (LC–MS) system (Thermo Scientific). Metabolite identification and quantification were performed with Compound Discoverer 3.3 software incorporating integrated tandem mass spectrometry spectral libraries. All metabolomic analyses were carried out by Shanghai Personalbio Technology Co. Ltd. (China).

### 2.14 Statistical Analysis

Data are expressed as mean ± standard deviation (SD). The Shapiro–Wilk test was applied to all datasets to assess normality of distribution. For data that passed the normality assumption, comparisons among groups were made using one‐way analysis of variance (ANOVA) followed by Tukey's post hoc test. For data that did not follow a Gaussian distribution, the Kruskal–Wallis test with Dunn's post hoc test was used instead. The Shapiro–Wilk test indicated that mNSS scores and Chiu's scores did not follow a normal distribution; therefore, the Kruskal–Wallis test with Dunn's post hoc test was used for these comparisons. All other datasets passed the normality assumption and were analyzed using one‐way ANOVA followed by Tukey's post hoc test. Tukey's post hoc test inherently controls the family‐wise error rate for multiple pairwise comparisons within individual analyses. For non‐metabolomic datasets analyzed by Kruskal–Wallis with Dunn's post hoc test, Bonferroni‐adjusted *p* values were applied to account for multiple comparisons.

Multivariate metabolomic analyses were conducted in R. All metabolomic data were log‐transformed and Pareto‐scaled prior to analysis. Principal component analysis (PCA) was first applied for unsupervised visualization of sample distribution. Supervised group discriminant was performed using partial least squares‐discriminant analysis (PLS‐DA) and orthogonal partial least squares‐discriminant analysis (OPLS‐DA). The optimal number of latent components was determined via cross‐validation, and model robustness was validated through permutation testing with 1000 random permutations. *R* ^2^ *X* and *R* ^2^ *Y* denote the explained variance of the *X* and *Y* matrices, respectively, whereas *Q* ^2^ reflects predictive ability. Metabolites meeting both adjusted *p* < 0.05 (Benjamini–Hochberg false discovery rate correction) and variable importance in projection (VIP) > 1 were regarded as significantly differential. FDR correction was applied to all metabolomic analyses to control for multiple testing. Hierarchical clustering was performed using pheatmap (version 1.0.12), and visualization was generated with ggplot2 (version 3.4.1). Pathway enrichment analysis of differential metabolites was carried out using Kyoto Encyclopedia of Genes and Genomes (KEGG) database with clusterProfiler (version 4.6.0).

## 3 Results

### 3.1 Lysozyme Reshapes Gut Microbial Metabolites and Attenuates Systemic Inflammation

**FIGURE 1:**
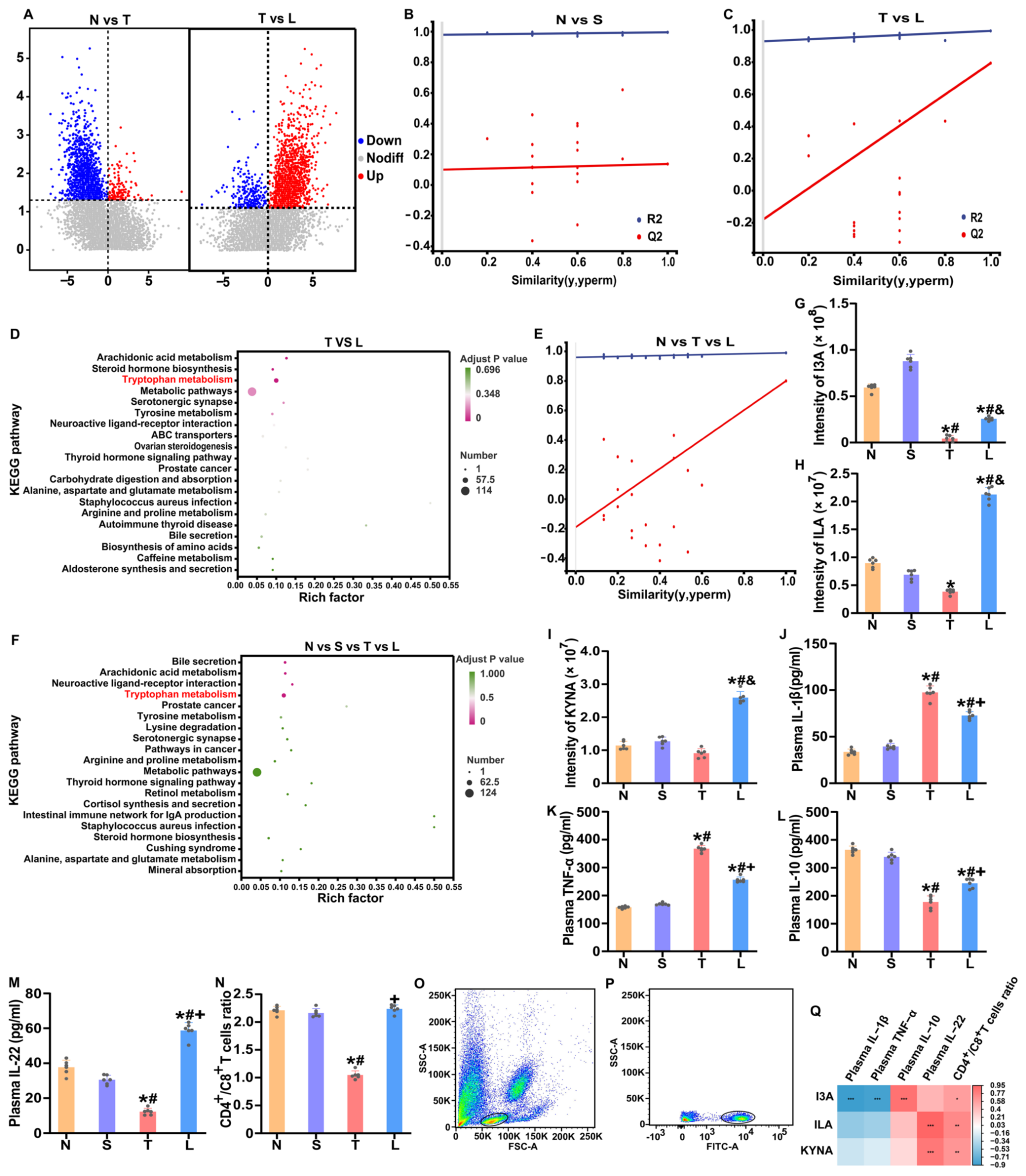
Lysozyme reshapes gut microbial metabolites and attenuates systemic inflammation after TBI. (A) Volcano plots for the normal versus TBI (N vs. T) and TBI versus lysozyme (T vs. L) comparisons. (B, C) OPLS‐DA score plots showing fecal metabolite profiles for the sham versus normal groups (B) and the TBI versus lysozyme groups (C). (D, F) KEGG pathway enrichment analysis of differential metabolites identified by untargeted fecal metabolomics. (E) PLS‐DA score plot showing separation among the Normal, TBI, and Lysozyme groups. (G–I) Relative fecal levels of the tryptophan metabolites indole‐3‐carboxaldehyde (I3A, G), indolelactic acid (ILA, H), and kynurenic acid (KYNA, I). (J–M) Plasma levels of IL‐1β (J), TNF‐α (K), IL‐10 (L), and IL‐22 (M). (N–P) Representative flow cytometry plots of CD4^+^ and CD8^+^ T cells in peripheral blood. (Q) Correlation heatmap showing the associations between metabolites (I3A, ILA, and KYNA) and inflammatory cytokines/CD4^+^/CD8^+^ T cell ratio. Data are presented as mean ± SD. Statistical comparisons among groups were performed by one‐way analysis of variance (ANOVA) followed by Tukey's post hoc test. *p < 0.05 versus N; ^#^ *p* < 0.05 versus S; ^+^ *p* < 0.05 versus T; ^&^ *p* < 0.05 versus T. L, lysozyme‐treated group; N, normal group; S, sham group; T, severe traumatic brain injury group. *n* = 6 per group.

Fecal metabolomic profiling revealed profound metabolic alterations after TBI and lysozyme treatment. Compared with the normal group, the TBI group exhibited 578 downregulated and 21 upregulated metabolites, whereas lysozyme treatment largely reversed this pattern (707 upregulated, 65 downregulated vs. TBI group; Figure 1A). OPLS‐DA confirmed no significant separation between normal and sham groups (Figure 1B) but clear divergence between TBI and lysozyme‐treated groups (Figure 1C). KEGG enrichment identified tryptophan metabolism as a significantly enriched pathway (Figure 1D). PLS‐DA demonstrated distinct clustering among normal, TBI, and lysozyme‐treated groups (Figure 1E), and KEGG analysis again highlighted tryptophan metabolism (Figure 1F). Within this pathway, I3A, indolelactic acid (ILA), and kynurenic acid (KYNA) were significantly increased by lysozyme treatment (Figure 1G–I).

TBI markedly increased plasma levels of the pro‐inflammatory cytokines IL‐1β and TNF‐α, both reduced by lysozyme. The anti‐inflammatory cytokines IL‐10 and IL‐22 were decreased after TBI and restored by lysozyme (Figure 1J–M). The CD4^+^/CD8^+^ T cell ratio was reduced after TBI and normalized by lysozyme (Figure 1N–P). I3A was inversely associated with IL‐1β and TNF‐α levels, while positively correlated with IL‐10 expression and the CD4^+^/CD8^+^ T cell ratio (Figure 1Q). Together, these findings indicate that lysozyme shifts gut microbial tryptophan metabolism toward a beneficial profile and alleviates systemic inflammation after TBI.

### 3.2 Lysozyme Alleviates Brain Injury and Neuroinflammation After TBI

**FIGURE 2:**
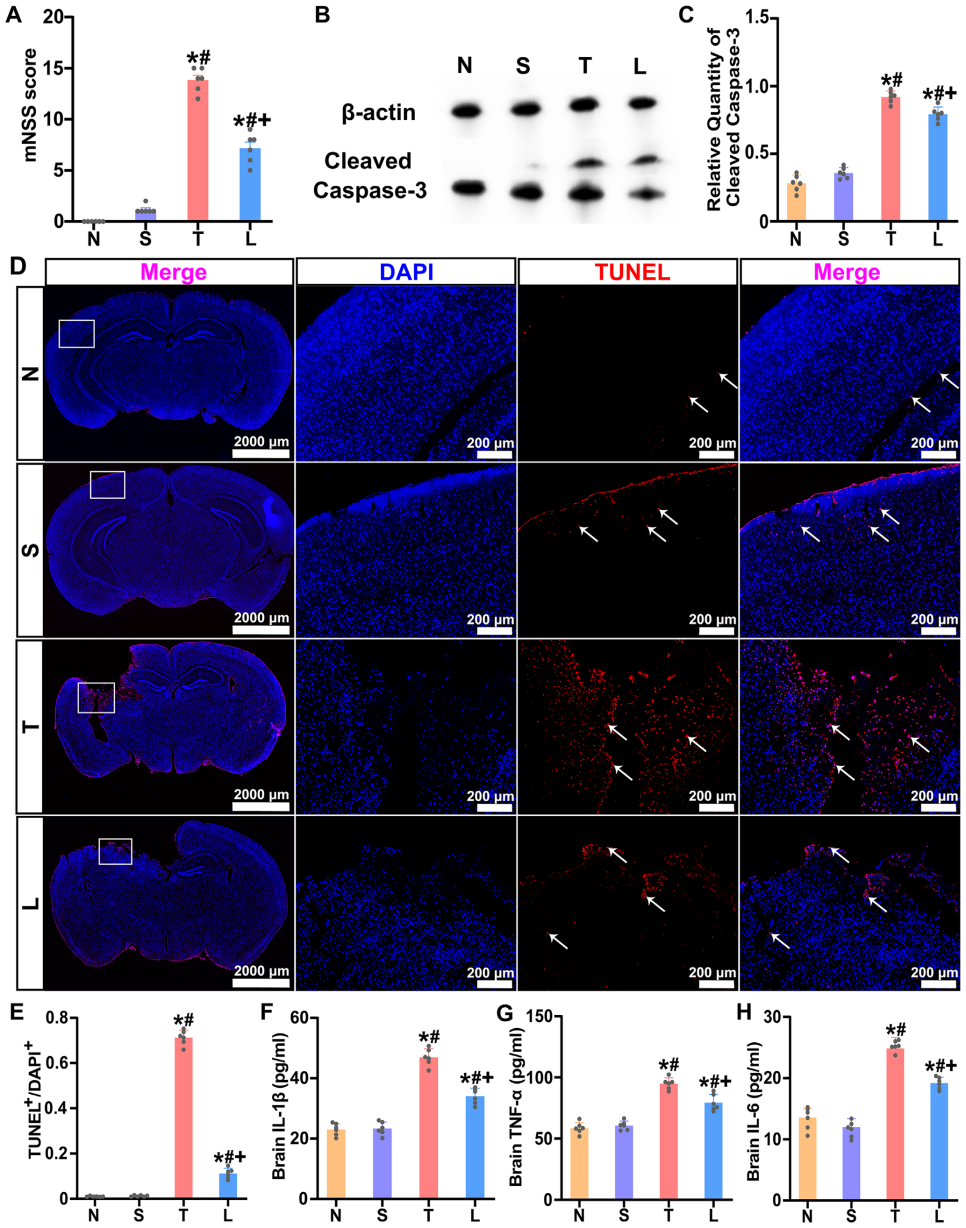
Lysozyme alleviates brain injury and neuroinflammation after TBI. (A) Modified Neurological Severity Score (mNSS) at Day 7 after TBI. (B, C) Western blot analysis of cleaved caspase‐3 expression in brain tissue (B) and corresponding quantification (C). (D, E) TUNEL staining of brain sections (D) and quantification of apoptotic cells (E). (F–H) Brain levels of the pro‐inflammatory cytokines IL‐1β (F), TNF‐α (G), and IL‐6 (H). Data are presented as mean ± SD. Modified Neurological Severity Score (mNSS) was analyzed by the Kruskal–Wallis test with Dunn's post hoc test; all other comparisons were performed by one‐way analysis of variance (ANOVA) followed by Tukey's post hoc test. *p < 0.05 versus N; ^#^ *p* < 0.05 versus S; ^+^ *p* < 0.05 versus T. Scale bar = 100 μm. L, lysozyme‐treated group; N, normal group; S, sham group; T, severe traumatic brain injury group. *n* = 6 per group.

Lysozyme improved neurological function, with lower mNSS scores at Day 7 (Figure 2A). Western blotting and TUNEL staining demonstrated reduced cleaved caspase‐3 expression and decreased apoptosis of cortical cells (Figure 2B–E, Data S1). Brain IL‐1β, TNF‐α, and IL‐6 were also lower in lysozyme‐treated mice (Figure 2F–H). These results suggest that lysozyme attenuates brain injury and neuroinflammation after TBI.

### 3.3 Lysozyme Is Associated With Alterations in Cerebral Tryptophan–Kynurenine Metabolism Favoring the Kynurenic Acid Branch

**FIGURE 3:**
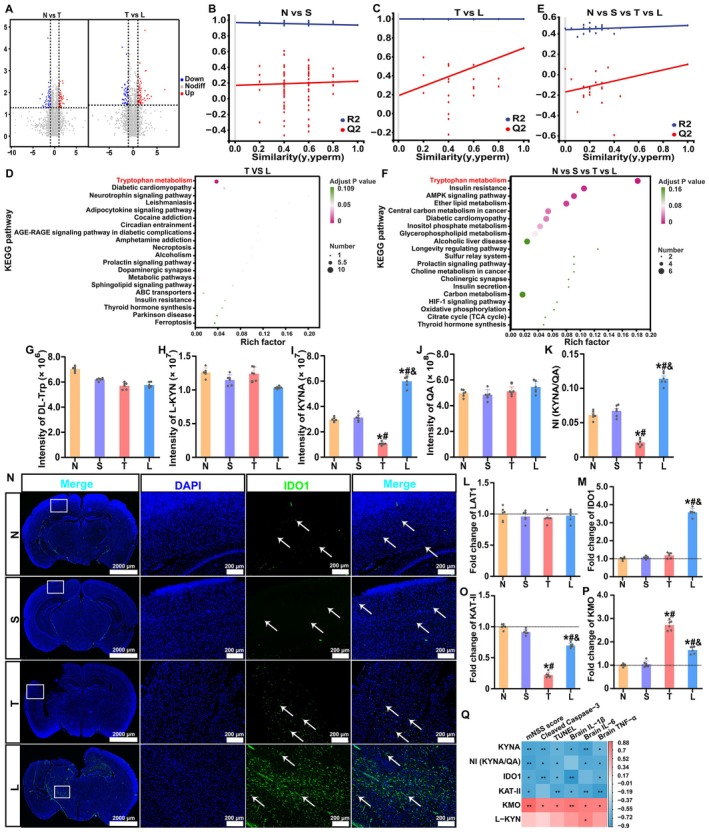
Lysozyme is associated with alterations in cerebral tryptophan–kynurenine metabolism favoring the kynurenic acid branch after TBI. (A) Volcano plots for the normal versus TBI (N vs. T) and TBI versus lysozyme (T vs. L) comparisons. (B, C) OPLS‐DA score plots of brain metabolites for the sham versus normal groups (B) and the TBI versus lysozyme groups (C). (D, F) KEGG pathway enrichment analysis of differential metabolites identified in brain tissue. (E) PLS‐DA score plot showing separation among groups. (G–J) Brain levels of DL‐tryptophan (G), L‐kynurenine (H), kynurenic acid (KYNA, I), and quinolinic acid (QA, J). (K) Neuroprotective index (NI), expressed as the KYNA/QA ratio. (L, M, O, P) RT‐PCR analysis of key metabolic enzymes, including LAT1 (L), IDO1 (M), KAT‐II (O), and KMO (P). (N) Immunofluorescence staining of IDO1. (Q) Correlation matrix showing the relationships between metabolic/enzyme markers and indices of injury and neuroinflammation. Data are presented as mean ± SD. Metabolomic data were analyzed by PLS‐DA and OPLS‐DA; differential metabolites were identified by adjusted *p* < 0.05 (Benjamini–Hochberg FDR correction) and VIP > 1. Gene expression and enzyme data were compared by one‐way analysis of variance (ANOVA) followed by Tukey's post hoc test. *p < 0.05 compared to N, ^#^ *p* < 0.05 compared to S, ^&^ *p* < 0.05 versus T. Q shows *p < 0.05, **p < 0.01, ***p < 0.001. Scale bar = 2000 or 200 μm. L, lysozyme‐treated group; N, normal group; S, sham group; T, severe traumatic brain injury group. *n* = 6 per group.

Brain metabolomic revealed significant enrichment of the tryptophan metabolism pathway following lysozyme treatment (Figure 3A–F). Brain KYNA levels and the KYNA/QA ratio (neuroprotective index) were elevated, whereas tryptophan, kynurenine, and QA levels remained unchanged (Figure 3G–K). Given that tryptophan metabolites cross the blood–brain barrier, we examined key cerebral kynurenine pathway enzymes. RT‐PCR analysis showed no difference in LAT1 expression (Figure 3L), whereas IDO1 and KAT‐II were increased and KMO was decreased in lysozyme‐treated mice (Figure 3M–P). KYNA, IDO1, and KAT‐II levels were inversely associated with neuroinflammatory markers, whereas KMO exhibited a positive correlation (Figure 3Q). Collectively, these data suggest lysozyme administration correlates with a shift in brain kynurenine metabolism favoring the KYNA arm, yet definitive causal proof via independent targeted metabolite quantification and functional interventions is absent from the current study.

### 3.4 The Protective Effects of Lysozyme on Intestinal and Systemic Inflammation Depend on the Gut Microbiota

**FIGURE 4:**
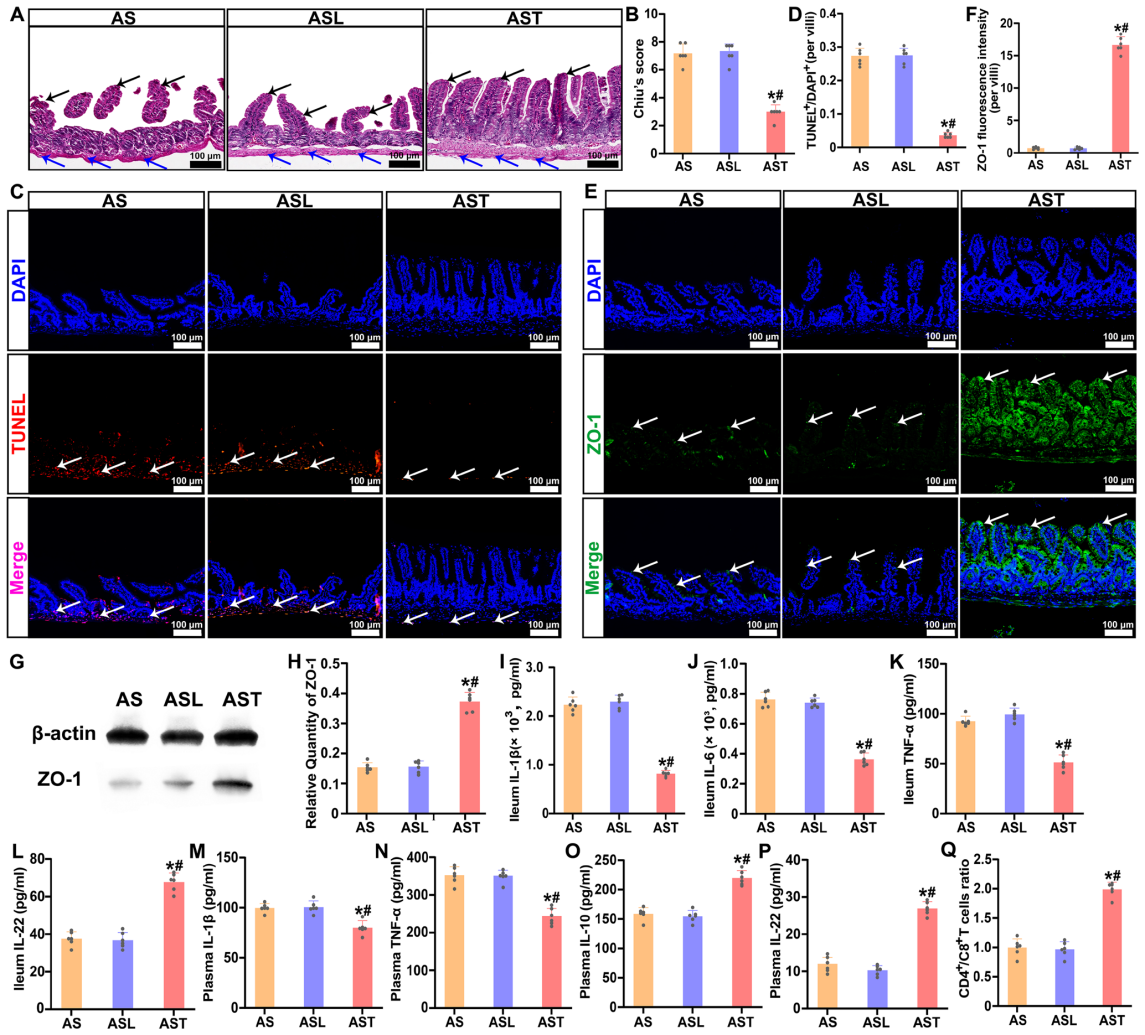
The beneficial effects of lysozyme on intestinal and systemic inflammation are dependent on the gut microbiota. (A) H&E staining of ileal tissue. (B) Chiu's score for ileal injury. (C) TUNEL staining of ileal tissue. (D) Quantification of TUNEL^+^/DAPI^+^ apoptotic cells per villus in the ileum. (E) Immunofluorescence staining of ZO‐1 in the ileum. (F) Quantification of ZO‐1 fluorescence intensity per villus. (G) Western blot analysis of ZO‐1 expression. (H) Quantification of Western blot results. (I–L) Ileal levels of IL‐1β (I), IL‐6 (J), TNF‐α (K), and IL‐22 (L). (M–P) Plasma levels of IL‐1β (M), IL‐6 (N), TNF‐α (O), and IL‐22 (P). (Q) CD4^+^/CD8^+^ T cell ratio in peripheral blood. Data are presented as mean ± SD. Chiu's score was analyzed by the Kruskal–Wallis test with Dunn's post hoc test; all other comparisons were performed by one‐way analysis of variance (ANOVA) followed by Tukey's post hoc test. *p < 0.05 versus AS, ^#^ *p* < 0.05 versus ASL. Scale bar = 100 μm. AS, ABX + sTBI + sterile water; ASL, ABX + sTBI + lysozyme; AST, ABX + sTBI + fecal microbiota transplantation (FMT). *n* = 6 per group.

In microbiota‐depleted mice, lysozyme treatment failed to ameliorate intestinal mucosal injury, as evidenced by unchanged Chiu's scores, apoptotic indices, and ZO‐1 expression levels (Figure 4A–H, Data S1). By contrast, FMT from lysozyme‐treated donors alleviated ileal injury, reduced apoptosis and pro‐inflammatory cytokines, and increased IL‐22 (Figure 4I–L). Similar effects were observed in plasma cytokines and the CD4^+^/CD8^+^ T cell ratio (Figure 4M–Q). These results indicate that the gut microbiota is required for the beneficial effects of lysozyme on intestinal integrity and systemic immune homeostasis.

### 3.5 Lysozyme‐Mediated Neuroprotection and Cerebral Metabolic Reprogramming Are Gut Microbiota Dependent

**FIGURE 5:**
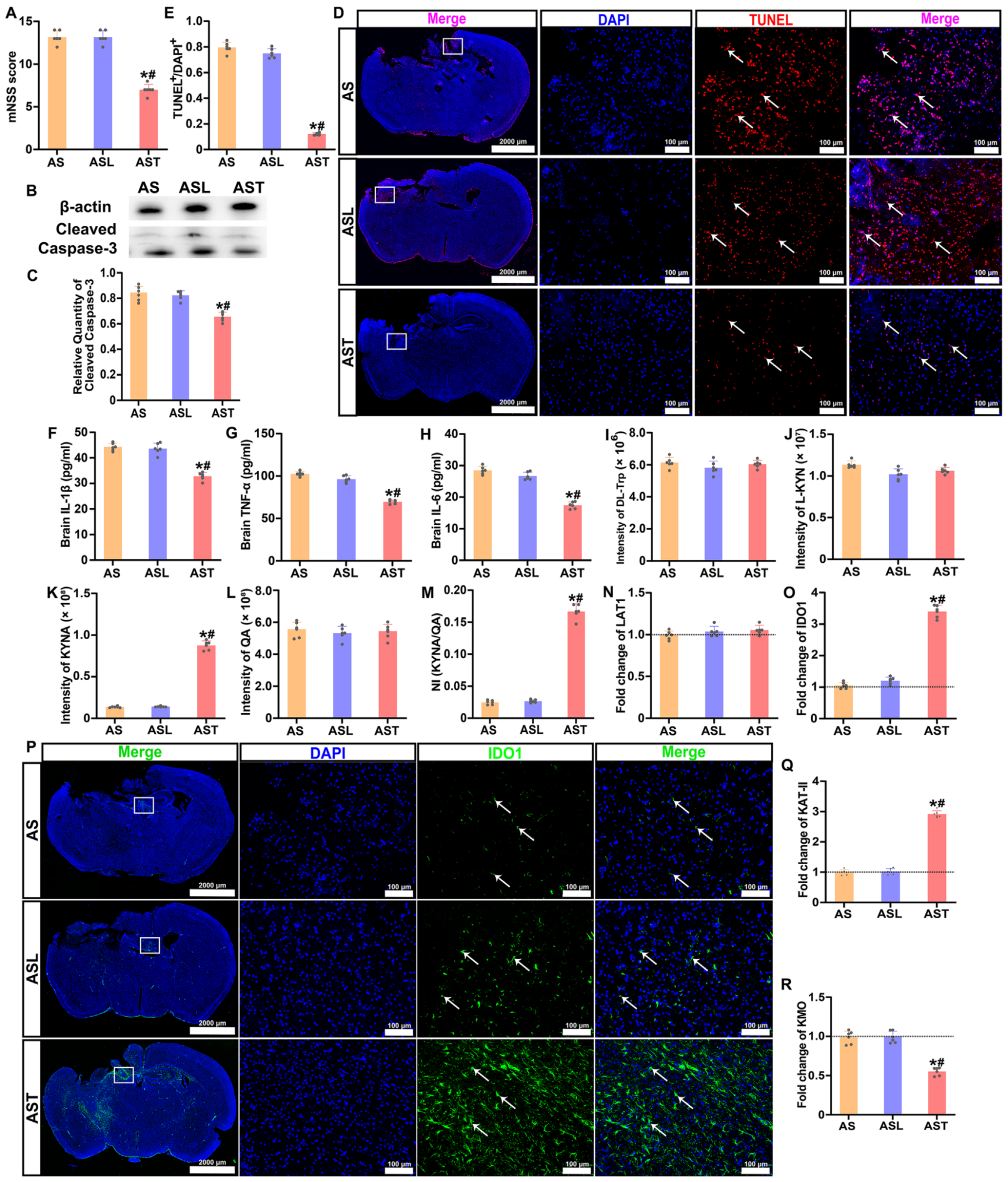
Lysozyme‐mediated neuroprotection and cerebral metabolic reprogramming are dependent on the gut microbiota. (A) Modified Neurological Severity Score (mNSS) at Day 7 after TBI. (B, C) Western blot analysis of cleaved caspase‐3 expression in brain tissue (B) and corresponding quantification (C). (D, E) TUNEL staining of brain sections (D) and quantification of apoptotic cells (E). (F–H) Brain levels of the pro‐inflammatory cytokines IL‐1β (F), TNF‐α (G), and IL‐6 (H). (I–L) Brain levels of DL‐tryptophan (I), L‐kynurenine (J), kynurenic acid (KYNA, K), and quinolinic acid (QA, L). (M) Neuroprotective index (NI), expressed as the KYNA/QA ratio. (N, O, Q, R) RT‐PCR analysis of key metabolic enzymes, including LAT1 (N), IDO1 (O), KAT‐II (Q), and KMO (R). (P) Immunofluorescence staining of IDO1. Data are presented as mean ± SD. Modified Neurological Severity Score (mNSS) was analyzed by the Kruskal–Wallis test with Dunn's post hoc test; all other comparisons were performed by one‐way analysis of variance (ANOVA) followed by Tukey's post hoc test. *p < 0.05 versus AS, ^#^ *p* < 0.05 versus ASL. Scale bar = 2000 or 100 μm. AS, ABX + sTBI + sterile water; ASL, ABX + sTBI + lysozyme; AST, ABX + sTBI + fecal microbiota transplantation (FMT). *n* = 6 per group.

FMT from lysozyme‐treated donors recapitulated the neuroprotective phenotype in microbiota‐depleted recipients, as evidenced by improved mNSS scores, attenuated brain apoptosis, and decreased pro‐inflammatory cytokine levels (Figure 5A–H, Data S1). FMT also increased brain KYNA and the KYNA/QA ratio, upregulated IDO1 and KAT‐II, and downregulated KMO (Figure 5I–R). These findings suggest that the gut microbiota is essential for lysozyme‐mediated neuroprotection and cerebral metabolic reprogramming in the context of TBI.

### 3.6 Enhanced Tryptophan–Kynurenine Pathway Activity in the CSF of Patients With Favorable Outcomes After TBI

**FIGURE 6:**
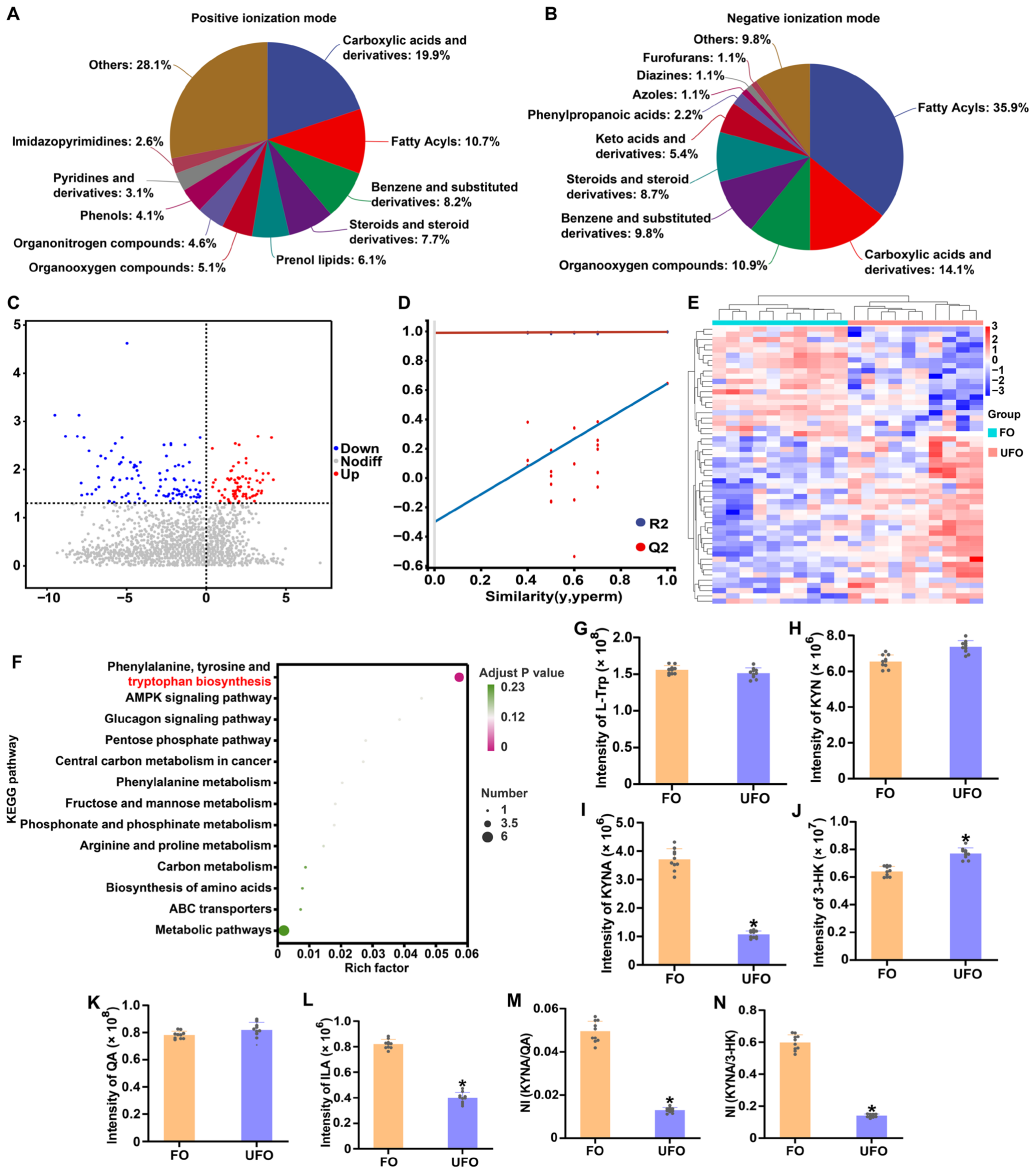
Enhanced tryptophan–kynurenine pathway activity in the CSF of patients with severe TBI and favorable outcomes. (A, B) Pie charts showing the chemical class distribution of metabolites detected in positive‐ion mode (A) and negative‐ion mode (B) by untargeted LC–MS. (C) Volcano plot of differential metabolites between the favorable‐ and unfavorable‐outcome groups. (D) OPLS‐DA score plot showing metabolic separation between outcome groups. (E) Heatmap and hierarchical clustering analysis of significantly differential metabolites, illustrating the metabolic patterns distinguishing the two outcome groups. (F) KEGG pathway enrichment analysis. (G–N) CSF levels of L‐tryptophan (L‐Trp, G), kynurenine (KYN, H), kynurenic acid (KYNA, I), 3‐hydroxykynurenine (3‐HK, J), quinolinic acid (QA, K), indolelactic acid (ILA, L), NI (KYNA/QA) ratio (NI, neuroprotective index, M), and NI (KYNA/3HK) ratio (NI, neuroprotective index, N). Data are presented as mean ± SD. Metabolomic data were analyzed by OPLS‐DA; differential metabolites were identified by adjusted *p* < 0.05 (Benjamini–Hochberg FDR correction) and VIP > 1. Comparisons between favorable‐ and unfavorable‐outcome groups were performed by unpaired *t*‐test for normally distributed data or Mann–Whitney *U* test for non‐normally distributed data, as determined by the Shapiro–Wilk test. *p < 0.05; *n* = 10 patients per group.

CSF metabolomic analysis identified 53 metabolites that differed between patients with favorable and unfavorable outcomes (Figure 6A–E). KEGG enrichment highlighted tryptophan metabolism as a significantly altered pathway (Figure 6F). Patients with favorable outcomes exhibited elevated CSF levels of KYNA and ILA, along with a higher KYNA/QA ratio, whereas 3‐hydroxykynurenine (3‐HK) levels were elevated in those with unfavorable outcomes (Figure 6G–N). These exploratory clinical findings suggest an association between a neuroprotective shift in tryptophan metabolism and improved TBI prognosis, although validation in larger cohorts is needed.

## 4 Discussion

This study suggests that oral lysozyme may initiate a gut‐to‐brain protective cascade after TBI by reshaping gut microbiota metabolism. Our data indicate that lysozyme administration was associated with enrichment of beneficial tryptophan metabolites—including I3A, ILA, and KYNA—reinforced intestinal barrier integrity, reestablished systemic immune homeostasis, and shifted cerebral kynurenine metabolism toward the neuroprotective KYNA branch while concurrently suppressing the neurotoxic QA pathway. Notably, these effects were abolished upon microbiota depletion and recapitulated by FMT, suggesting a critical dependence on the gut microbiota. The concordance between preclinical findings and CSF metabolomic profiles in patients with severe TBI provides preliminary support for the translational relevance of this mechanism, although the small clinical sample size warrants cautious interpretation.

Lysozyme hydrolyzes peptidoglycan with preferential activity against Gram‐positive bacteria, while Gram‐negative organisms are partially shielded by their outer membrane [26]. Beyond bactericidal effects, lysozyme possesses non‐lytic immunomodulatory functions and is released by Paneth cells via secretory autophagy, so its protective effects arise from selective antimicrobial action, pathobiont suppression, and immune engagement [27, 28]. Our previous 16S rRNA sequencing showed that oral lysozyme restored beneficial genera including *Oscillospira*, *Ruminococcus*, *Alistipes*, *Butyricicoccus*, and *Lactobacillus* [18]. These compositional findings indicate that lysozyme selectively enriches beneficial genera while reducing pathobionts. Building on this, the present study suggests that lysozyme extends beyond mere compositional remodeling to reprogram the overall metabolic output of the microbiota, steering the microbial ecosystem toward a more stable and host‐beneficial functional state. Compared with strategies relying solely on exogenous probiotic supplementation, modulation of endogenous microbial function may offer greater robustness and sustainability. Microbial metabolites, particularly indole derivatives and short‐chain fatty acids, are critical mediators of host–microbe communication [29, 30]. The increased I3A and ILA are of particular interest, as these AhR ligands promote IL‐22 production and tight junction integrity [31]. Consistent with this proposed mechanism, lysozyme treatment elevated I3A and ILA levels, upregulated ZO‐1 expression, enhanced IL‐22 production, and improved intestinal barrier function—collectively supporting a microbiota–AhR signaling axis that constrains intestinal permeability and attenuates downstream systemic inflammation.

This study delineates a spatiotemporally coordinated protective cascade from gut to brain. At the intestinal level, lysozyme enhanced barrier integrity and reduced injury, reflected by improved histology, reduced apoptosis, and increased ZO‐1. This is likely mediated partly by microbiota‐derived AhR ligands that reinforce epithelial homeostasis [32]. Because intestinal barrier dysfunction is an early consequence of TBI, barrier preservation may represent the first line of defense against dissemination of gut‐derived inflammatory signals.

At the systemic level, lysozyme restored immune homeostasis, reducing pro‐inflammatory cytokines, increasing IL‐10 and IL‐22, and normalizing the CD4^+^/CD8^+^ T cell ratio. TBI causes simultaneous hyperinflammation and immune suppression [33]; lysozyme counteracts this through several mechanisms. First, improved barrier function reduces translocation of microbial products, dampening systemic immune activation [34]. Second, intestinal AhR activation promotes regulatory immune programs including T cell differentiation and cytokine production [35]. Third, the shift toward an anti‐inflammatory milieu favors adaptive immune recovery. Restoring systemic immune balance may attenuate neuroinflammation, reduce secondary infection risk, and support neural repair [36].

At the cerebral level, lysozyme treatment was associated with remote metabolic alterations in the kynurenine pathway, increasing KYNA and the KYNA/QA ratio while upregulating IDO1 and KAT‐II and downregulating KMO—a shift toward the neuroprotective branch. KYNA is an endogenous antagonist of N‐methyl‐D‐aspartate receptors and α7 nicotinic acetylcholine receptors and has well‐recognized anti‐excitotoxic and anti‐inflammatory properties [37]. In contrast, QA and other downstream metabolites of the neurotoxic branch contribute to excitotoxicity, oxidative stress, and neuronal injury. Correlation analyses further support the functional relevance of this pathway: KYNA, IDO1, and KAT‐II were inversely associated with neuroinflammatory markers, whereas KMO exhibited the opposite pattern. The molecular mediators linking gut‐derived tryptophan metabolites to altered cerebral enzyme expression remain incompletely characterized, though several plausible signaling pathways can be proposed. First, tryptophan metabolites cross the blood–brain barrier via LAT1 and other amino acid transporters; upon entering systemic circulation, gut‐originated metabolites may act directly to modulate brain kynurenine pathway enzymes or serve as their substrates [10, 38]. Second, lysozyme‐mediated restoration of systemic immune homeostasis may indirectly regulate cerebral IDO1, KAT‐II, and KMO expression through circulating inflammatory cytokines and peripheral immune cell infiltration into the central nervous system [39, 40]. Third, vagal afferent nerves and enteric neural circuits transmit microbiota‐dependent metabolic signals to the brain via the gut–brain axis [41]. These pathways likely act synergistically rather than independently to mediate gut–brain crosstalk. The complete abrogation of lysozyme efficacy following microbiota depletion, together with phenotype restoration by FMT, strongly supports this interpretation.

The microbiota depletion and FMT experiments provide evidence supporting the gut microbiota as a critical mediator of lysozyme's effects. Antibiotic depletion abolished lysozyme's benefits on intestinal injury, systemic inflammation, and brain pathology. Although antibiotics may have direct immunomodulatory effects beyond microbial depletion, FMT‐mediated reproduction of protective phenotypes supports a microbiota‐dependent mechanism. Transplantation of fecal microbiota from lysozyme‐treated donors recapitulated most protective phenotypes in recipients, indicating that lysozyme itself is unlikely to function as a direct neuroprotective agent. Instead, its efficacy appears to rely on microbial community remodeling and associated metabolic shifts that collectively restore immune and neurochemical homeostasis [9, 11, 18, 42]. While the data suggest microbiota dependence and strong metabolic correlations, direct causal validation through metabolite‐specific interventions (e.g., KYNA supplementation or KMO inhibition) is needed. Nonetheless, these findings broaden lysozyme's identity from a classical antimicrobial enzyme to a microbiota‐modulating agent with neuroprotective potential, supporting the therapeutic rationale for targeting the gut–brain–microbiota axis in TBI.

Translational relevance is strengthened by paired CSF metabolomics in patients with severe TBI. Favorable outcomes correlated with higher CSF KYNA and ILA levels and an elevated KYNA/QA ratio, whereas 3‐HK, a neurotoxic KMO‐dependent intermediate, was elevated in patients with poor outcomes. Since 3‐HK drives oxidative stress and mitochondrial dysfunction [43], these data suggest that adverse TBI prognosis reflects both inadequate KYNA branch activation and enhanced neurotoxic kynurenine pathway flux. These clinical data are consistent with our animal findings and suggest the KYNA/QA ratio as a candidate prognostic indicator that requires rigorous follow‐up research; notably, the small sample size and single‐time‐point CSF sampling preclude definitive conclusions about its utility as a clinical biomarker. However, the small sample size limits generalizability; validation in larger, independent cohorts with longitudinal sampling is needed. Kynurenine pathway dysregulation has been recognized across acute neurological conditions including ischemic stroke, intracerebral hemorrhage, post‐cardiac arrest brain injury, and central nervous system infections [44, 45], where elevated QA and 3HK with reduced KYNA associate with worse outcomes. This convergence underscores the translational relevance of targeting tryptophan metabolism and positions our findings within a broader neurocritical care context. Only male mice were recruited for in vivo experiments to eliminate confounding effects from sex hormones, which are known to regulate immune reactivity, gut microbiota, and post‐TBI neuropathology [46, 47]. Nevertheless, accumulating evidence has documented profound sex‐dependent differences across all these biological domains. Females harbor distinct gut microbial communities with significant disparities in microbial diversity and taxonomic abundance relative to males [48, 49]. Sex also shapes immune function: females typically exhibit stronger innate and adaptive immune responses, which may alter the trajectory of neuroinflammatory following TBI [50, 51]. Furthermore, preclinical TBI models have identified sex‐specific neuropathological phenotypes, including exacerbated neuronal loss and astrogliosis in female animals, alongside divergent neuroinflammatory and functional outcomes [51, 52]. Recent research additionally suggests that gut‐targeted interventions, including probiotics and FMT, elicit sexually dimorphic neuroprotective effects after TBI, highlighting biological sex as a critical confounding variable [53]. Collectively, the results presented herein are primarily applicable to male subjects, and future studies incorporating both male and female animals are essential to determine whether the microbiota and metabolic alterations we identified are sex‐specific or broadly conserved.

### 4.1 Strengths and Limitations

This study has several notable strengths. First, it systematically delineates a mechanistic axis linking lysozyme‐induced gut microbiota remodeling to metabolic reprogramming, systemic immune regulation, and neuroprotection. Second, it uses both microbiota depletion and FMT to establish causality, rather than relying solely on associative observations. Third, it integrates preclinical and clinical metabolomic data, thereby enhancing translational relevance.

Several inherent limitations should be noted to appropriately contextualize the present clinical findings. First, we recruited only 10 matched patient pairs for CSF analysis, which confines all clinical outcomes to exploratory preliminary observations rather than definitive clinical conclusions. The purported biomarker value of the KYNA/QA ratio thus necessitates validation within larger, independent patient cohorts to support future translational development. Moreover, CSF was obtained at a single time point, precluding temporal analysis; longitudinal sampling would be needed to characterize the dynamic evolution of metabolomic changes. Second, all animal experiments were performed solely on male mice, limiting the translational generalizability of our findings to female TBI patients. Third, we did not perform serum metabolomic profiling, which prevents us from providing direct evidence of peripheral metabolite trafficking into the brain; nevertheless, our indirect biochemical data and FMT experiments lend preliminary support to this mechanistic model. Fourth, the causal relationship between specific metabolites (e.g., KYNA) and neuroprotection remains correlational, requiring direct metabolite intervention studies. Fifth, antibiotic‐mediated microbiota gut depletion may induce unrecognized off‐target immunomodulatory effects, and the intermediate molecular factors connecting peripheral metabolic shifts to cerebral kynurenine pathway enzyme expression remain uncharacterized. Finally, all experimental endpoints were assessed at post‐injury Day 7, corresponding to the subacute TBI phase. Future longitudinal studies incorporating serial sampling time points (e.g., Days 1, 3, 7, 14, 28) are needed to delineate the dynamic temporal kinetics of tryptophan–kynurenine pathway dysregulation. Future investigations should validate these findings in larger multicenter cohorts with longitudinal CSF sampling, test combinatorial therapeutic strategies involving KMO inhibition and KYNA supplementation, and evaluate whether these protective mechanisms extend to female subjects and other acute brain injury characterized by disrupted tryptophan–kynurenine metabolism.

## 5 Conclusion

This study suggests that oral lysozyme may attenuate TBI‐induced neuroinflammation and brain injury, potentially through gut microbiota‐dependent reprogramming of tryptophan metabolism. Lysozyme was associated with reinforcement of intestinal barrier integrity, restoration of systemic immune homeostasis, and redirection of cerebral kynurenine metabolism toward the KYNA branch while suppressing QA‐mediated neurotoxicity, consistent with a coordinated gut–systemic–brain protective cascade. These effects were abolished by microbiota depletion and recapitulated by FMT, supporting a critical role for the gut microbiota. Clinical CSF metabolomics further revealed that favorable TBI outcomes were associated with higher KYNA levels and an elevated KYNA/QA ratio, mirroring the preclinical findings. These results support further investigation of lysozyme as a candidate microbiota‐targeted therapeutic strategy. The KYNA/QA ratio merits further longitudinal evaluation in larger clinical cohorts to confirm its utility as a tentative prognostic indicator for TBI.

## Author Contributions

**Zhuoying Du:** data curation, writing – original draft, writing – review and editing. **Haijun Yao:** data curation, writing – original draft, writing – review and editing. **Caihua Xi:** formal analysis, data curation, writing – review and editing. **Qiang Yuan:** formal analysis, writing – review and editing. **Pengfei Fu:** formal analysis, writing – review and editing. **Jin Hu:** funding acquisition, supervision, writing – review and editing. **Gang Wu:** conceptualization, methodology, project administration, supervision, writing – review and editing. **Weijian Yang:** conceptualization, methodology, data curation, funding acquisition, writing – review and editing.

## Funding

This work was supported by the National Natural Science Foundation of China (grant numbers 82401618, 82471407, 82171382) and Research Foundation of Huashan Hospital, affiliated to Fudan University (grant number 2021QD026).

## Ethics Statement

For the animal experiments: All experimental procedures were approved by the Ethics Committee of Huashan Hospital, Fudan University (approval no. 2024‐HSYY‐438; March 1, 2024) and conducted in strict accordance with the guidelines for animal experimental research. For the collection of CSF from patients with TBI: This study was conducted in accordance with the Declaration of Helsinki and approved by the Ethics Committee of Huashan Hospital, Fudan University (approval no. 2022‐062; February 17, 2022).

## Consent

Informed consent was obtained from the patients' legal representatives for the collection of CSF samples for metabolomics analysis.

## Conflicts of Interest

The authors declare no conflicts of interest.

## Supporting information


**Data S1:** Original blot images for Western blot analysis of Figures 2B, 4G, and 5B.

**Table S1:** Baseline clinical characteristics of the TBI patients.

## Data Availability

The data that support the findings of this study are available from the corresponding author upon reasonable request.
