# Supplementary figures and images for: Oral Lysozyme Attenuates Neuroinflammation and Brain Injury After Traumatic Brain Injury Through Gut Microbiota‐Dependent Reprogramming of Tryptophan Metabolism
Source: CNS Neurosci Ther. 2026 Jul 10;32(7):e71025. doi: 10.1002/cns.71025 (PMC13353188; doi:10.1002/cns.71025)

Figure 2B

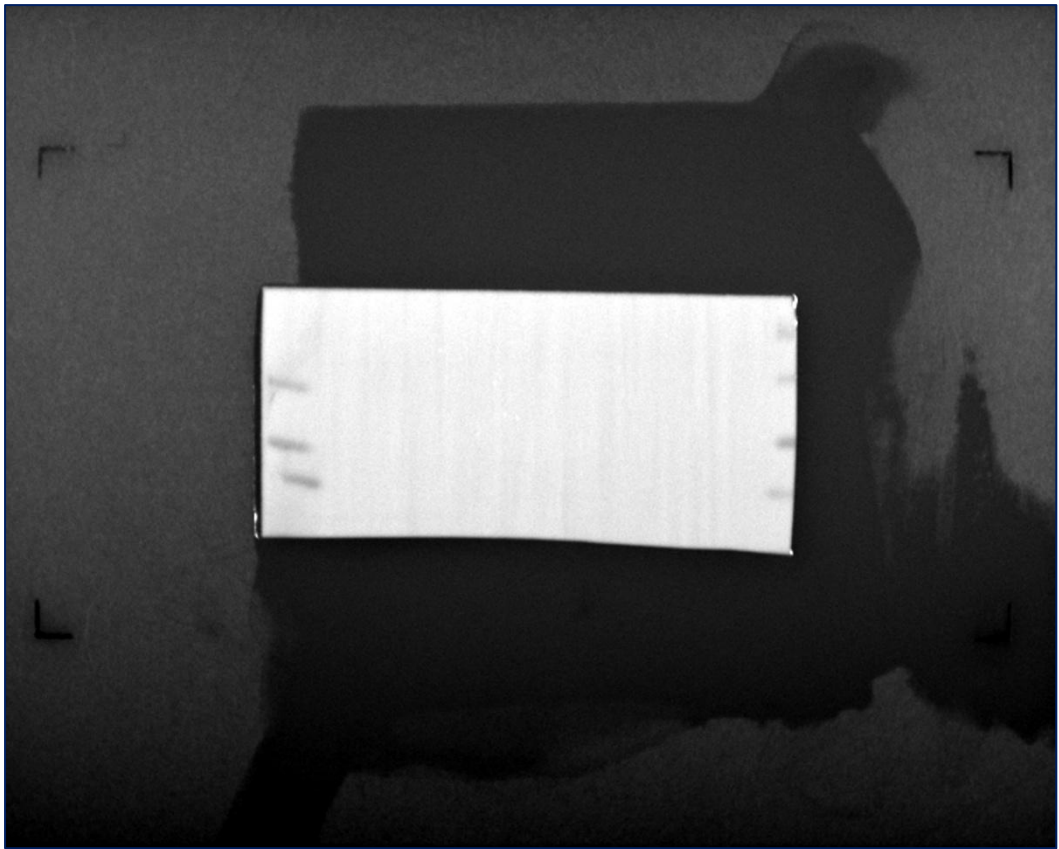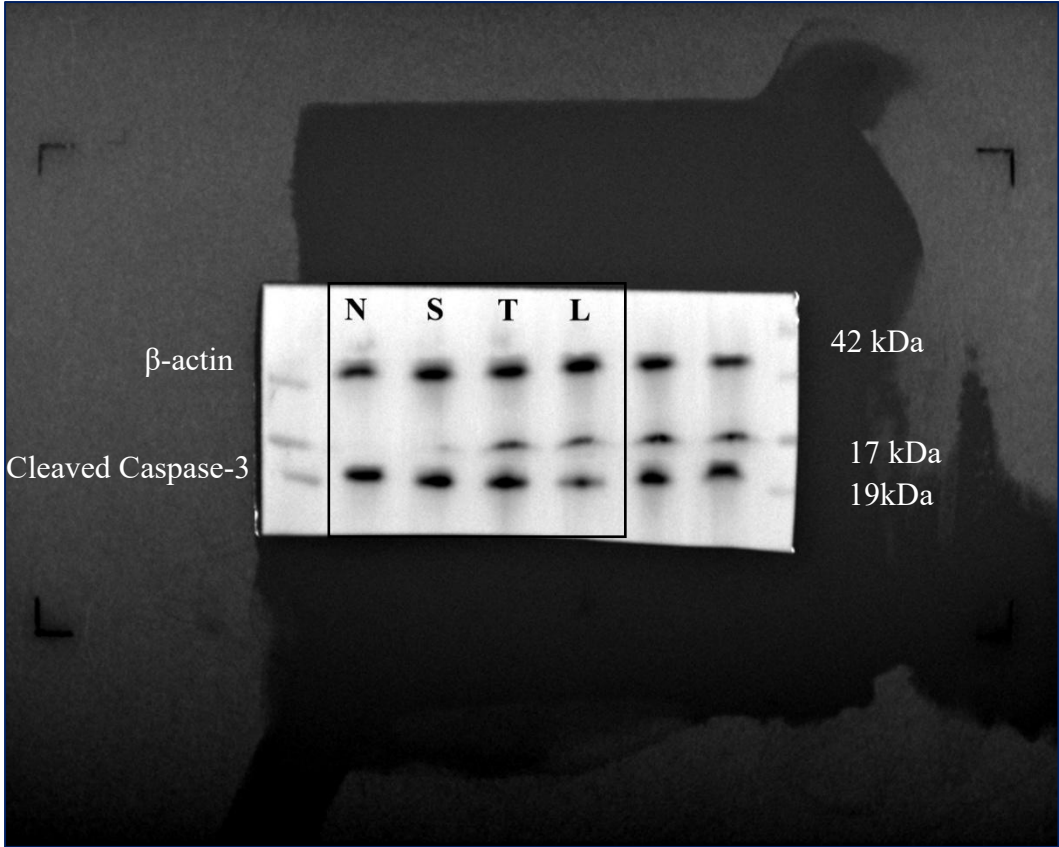

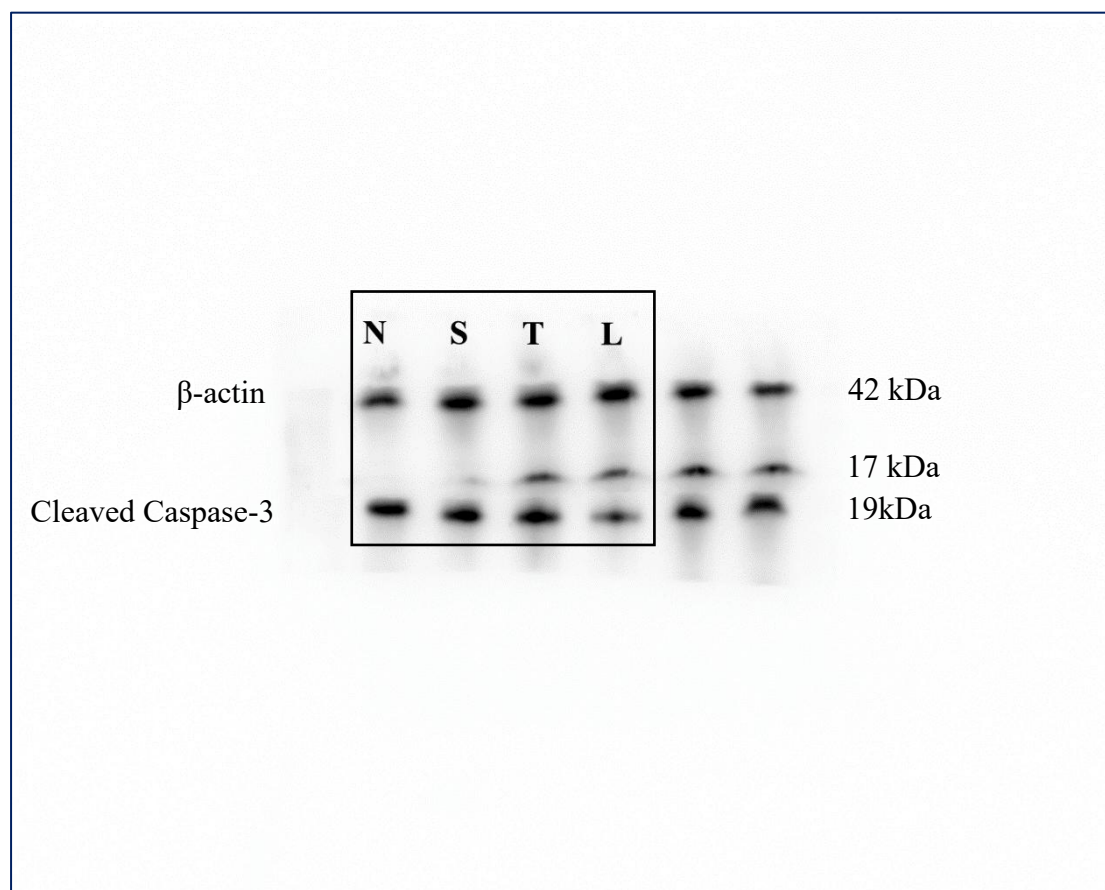

**Figure 4G**

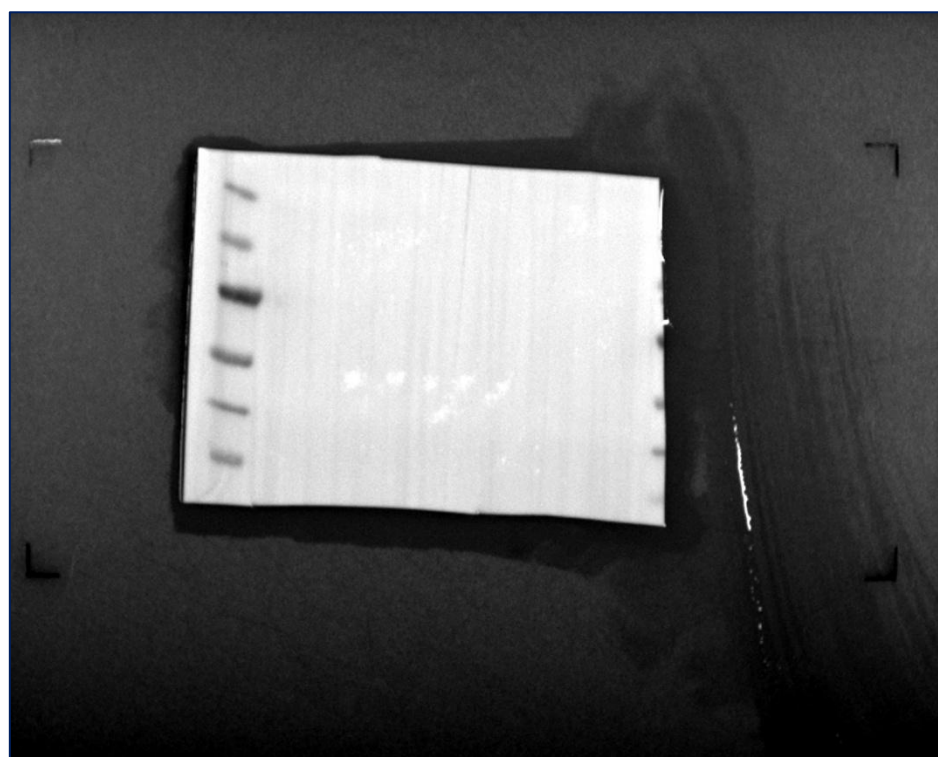

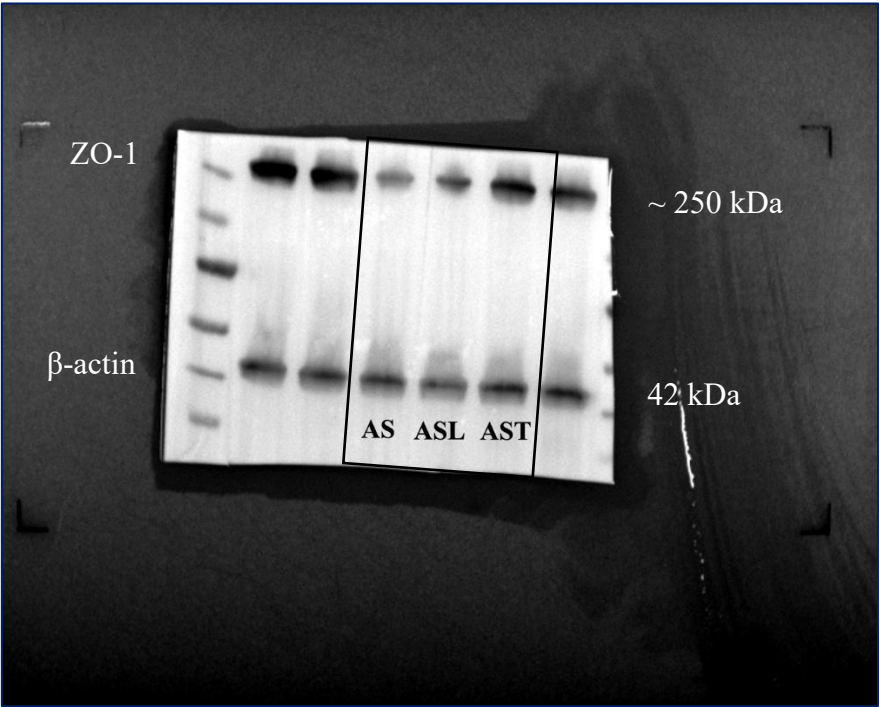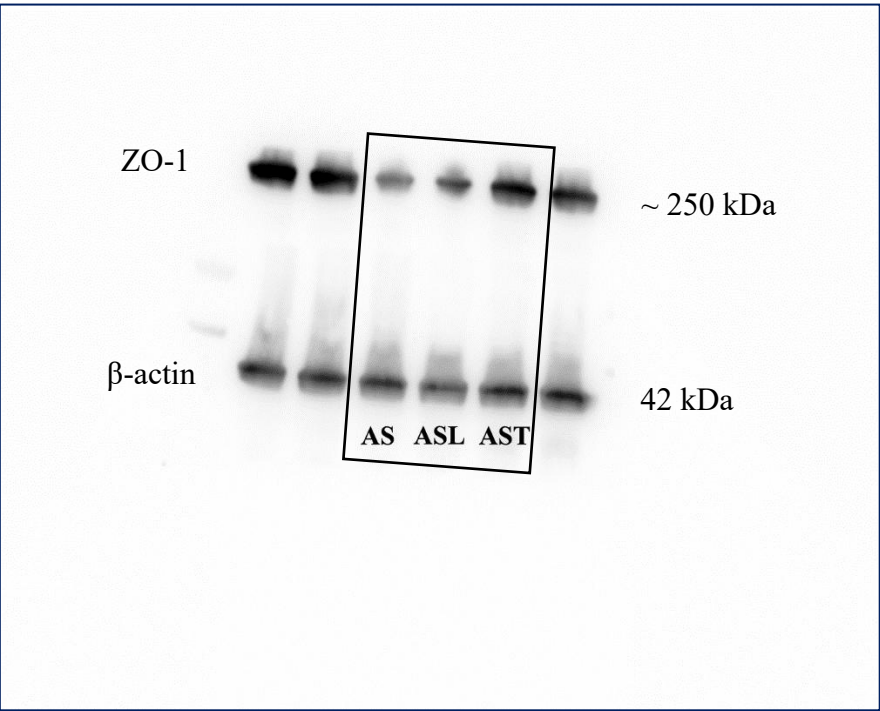

**Figure 5B**

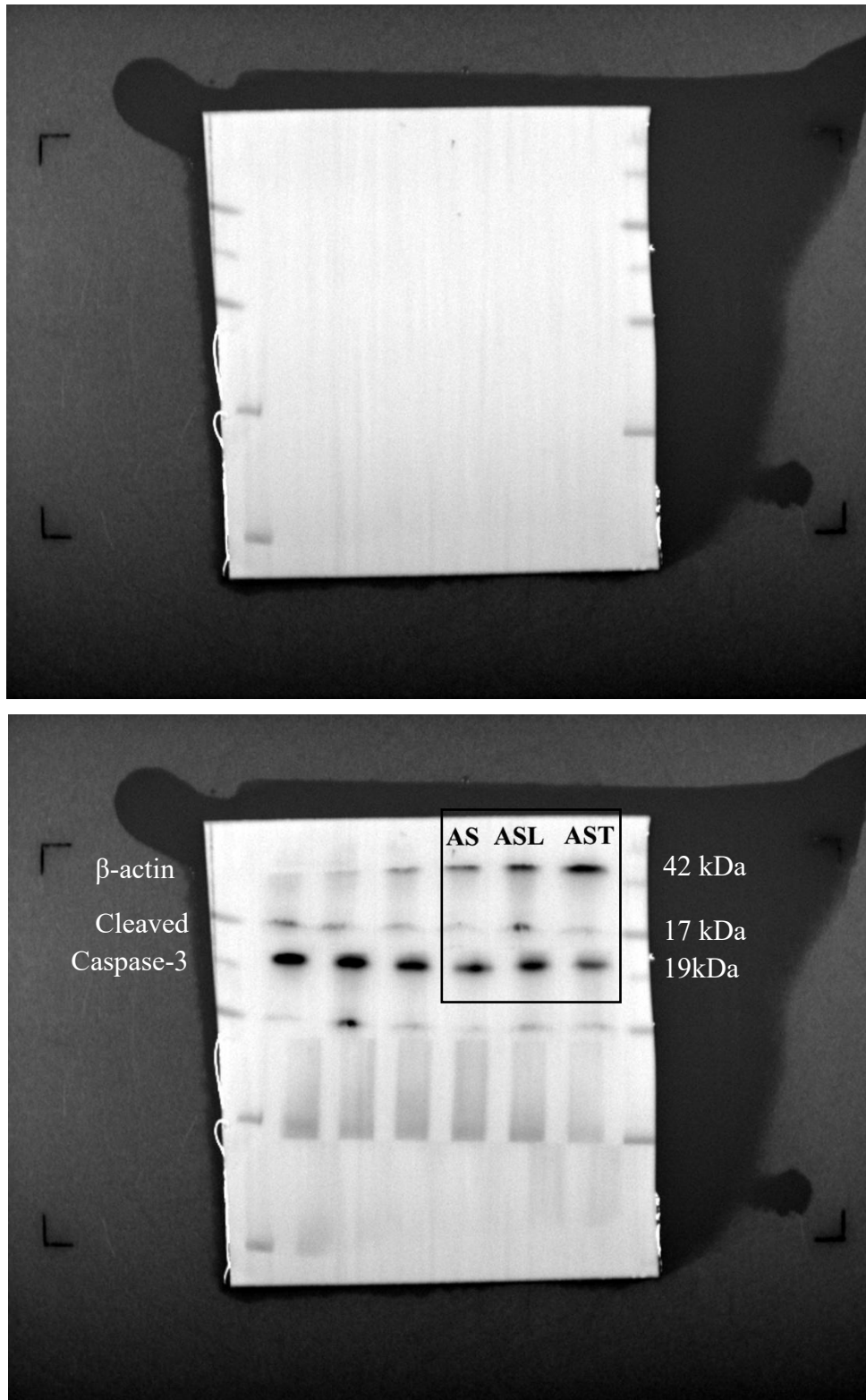

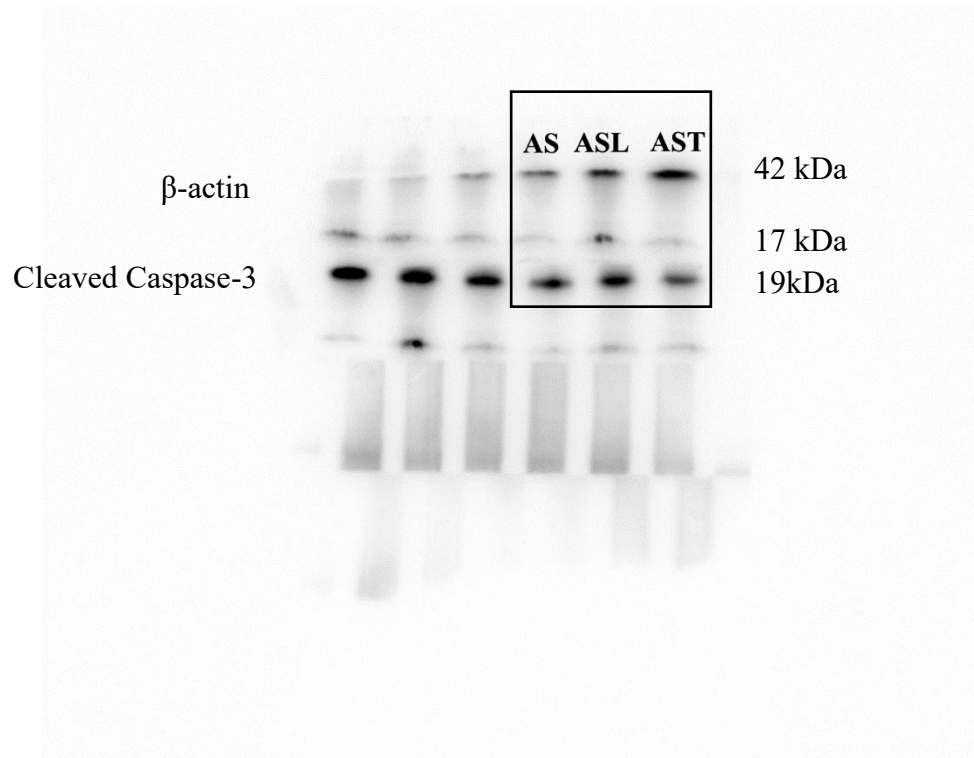

Supplement: Supplementary file 1 — Data S1: Original blot images for Western blot analysis of Figures 2B, 4G, and 5B. [file CNS-32-e71025-s001.pdf]
