# Supplementary material for: Oral Lysozyme Attenuates Neuroinflammation and Brain Injury After Traumatic Brain Injury Through Gut Microbiota‐Dependent Reprogramming of Tryptophan Metabolism
Source: CNS Neurosci Ther. 2026 Jul 10;32(7):e71025. doi: 10.1002/cns.71025 (PMC13353188; doi:10.1002/cns.71025)
Supplement: Supplementary file 2 — Table S1: Baseline clinical characteristics of the TBI patients. [file CNS-32-e71025-s002.docx]

Table S1. Baseline clinical characteristics of the TBI patients

|  | Unfavorable outcome | Favorable outcome | *P* |
| --- | --- | --- | --- |
| **Age (years), Mean ± SD** | 53.0 ± 10.3 | 53.9 ± 9.9 | 0.73 |
| **Sex-**Male, N (%) | 10 (100%) | 10 (100 %) |  |
| **TBI cause**-Road traffic accident, N (%) | 10 (100%) | 10 (100%) |  |
| **GCS score, Median (IQR)** | 5 [4, 6] | 5 [4, 6] | 1.00 |
| GCS-E, Median (IQR) | 1 [1, 2] | 1 [1, 1] | 0.26 |
| GCS-V, Median (IQR) | 1 [1, 1] | 1 [1, 1] | 0.71 |
| GCS-M, Median (IQR) | 2 [1, 3] | 3 [2, 3] | 0.41 |
| **Rotterdam CT score (Sum), Median (IQR)** | 5 [5, 6] | 6 [5, 6] | 0.71 |
| Basal cisterns, Median (IQR) | 2 [1, 2] | 2 [1, 2] | 1.00 |
| Midline shift, Median (IQR) | 1 [1, 1] | 1 [1, 1] | 1.00 |
| Epidural mass lesion, Median (IQR) | 1 [1, 1] | 1 [1, 1] | 0.71 |
| Intraventricular blood or tSAH, Median (IQR) | 1 [1, 1] | 1 [1, 1] | 1.00 |
| **Antithrombosis therapy, N (%)** | | |  |
| No | 10 (100%) | 100 (100%) |  |
| **Coagulation parameters** |  |  |  |
| International normalized ratio | 1.09 ± 0.25 | 1.08 ± 0.15 | 0.92 |
| Prothrombin time (s) | 12.05 ± 5.69 | 12.14 ± 1.52 | 0.96 |
| Activated partial thromboplastin time (s) (s) | 25.66 ± 5.26 | 24.23 ± 4.29 | 0.51 |
| Fibrinogen (g/L) | 2.48 ± 1.32 | 2.59 ± 1.11 | 0.84 |
| Thrombin time (s) | 17.92 ± 3.02 | 17.21 ± 2.46 | 0.57 |
| D-dimer | 25.38 ± 26.41 | 23.78 ± 23.52 | 0.89 |
| **Hematologic parameters** |  |  |  |
| White blood cell (10^9/L) | 16.36 ± 5.65 | 15.45 ± 5.41 | 0.72 |
| Neutrophil counts (10^9/L) | 13.54 ± 5.01 | 13.09 ± 4.57 | 0.84 |
| Percentage of neutrophils (%) | 81.87 ± 12.05 | 84.74 ± 10.28 | 0.57 |
| Lymphocyte count (10^9/L) | 1.68 ± 1.50 | 1.59 ± 1.49 | 0.89 |
| Percentage of lymphocytes (%) | 11.01 ± 10.24 | 10.84 ± 9.74 | 0.97 |
| Red blood cell (10^12/L) | 4.21 ± 0.63 | 4.39 ± 0.56 | 0.51 |
| Hemoglobin (g/L) | 131.47 ± 17.85 | 132.11 ± 16.57 | 0.93 |
| Hematocrit (%) | 38.90 ± 5.08 | 39.13 ± 4.42 | 0.92 |
| Platelet (10^9/L) | 182.04 ± 61.74 | 181.27 ± 53.35 | 0.97 |
| **Serum electrolytes** |  |  |  |
| Sodium (Na) (mmol/L) | 143.42 ± 5.85 | 141.57 ± 5.78 | 0.49 |
| Potassium (K) (mmol/L) | 3.83 ± 0.40 | 3.87 ± 0.49 | 0.84 |
| Calcium (Ca) (mmol/L) | 1.99 ± 0.27 | 2.01 ± 0.31 | 0.88 |
| Phosphorus (P) (mmol/L) | 0.89 ± 0.44 | 0.89 ± 0.39 | 1.00 |
| **Total protein (g/L)** | 54.39 ± 8.13 | 55.54 ± 6.86 | 0.74 |
| **Albumin (g/L)** | 41.34 ± 5.24 | 42.57 ± 5.83 | 0.63 |

TBI: Traumatic Brain Injury, GCS: Glasgow Coma Scale, GCS-M: Glasgow Coma Scale Motor Score, tSAH: traumatic subarachnoid hemorrhage
